# Seasonal Occurrence of Potato Psyllid (*Bactericera Cockerelli*) and Risk of Zebra Chip Pathogen (*Candidatus Liberibacter Solanacearum*) in Northwestern New Mexico

**DOI:** 10.3390/insects11010003

**Published:** 2019-12-19

**Authors:** Koffi Djaman, Charles Higgins, Shantel Begay, Komlan Koudahe, Samuel Allen, Kevin Lombard, Michael O’Neill

**Affiliations:** 1 Department of Plant and Environmental Sciences, New Mexico State University, Agricultural Science Center at Farmington, P.O. Box 1018, Farmington, NM 87499, USA; samallen@nmsu.edu (S.A.); klombard@nmsu.edu (K.L.); moneill@nmsu.edu (M.O.); 2 Higgins Farms Inc., 4220 N. Crescent Ave, Farmington, NM 87401, USA; higginsfarms@comcast.net; 3 Wilbur-Ellis Co., 9813 NM-371, Farmington, NM 87401, USA; SBegay@wilburellis.com; 4 ADA Consulting Africa, Lomé 07 BP 14284, Togo; koudahe_komlan@yahoo.fr

**Keywords:** potato psyllid, population dynamics, zebra chip pathogen

## Abstract

Potato psyllid (*Bactericera cockerelli*) is one of the most important pests in potatoes (*Solanum tuberosum* L.) due to its feeding behavior and the transmission of a bacterium (*Candidatus* Liberibacter solanacearum) that causes zebra chip disease, altering the quality of the potato tuber and the fried potato chip or french fry. This pest is thus a threat to the chip potato industry and often requires preventive measures including the use of costly insecticides. The objectives of this research were to monitor the variation in *B. cockerelli* adult abundance and to evaluate the risk of zebra chip disease in northwestern New Mexico, USA. Yellow sticky traps were used to collect the pest at the Agricultural Experiment Station at Farmington, NM and in nearby commercial fields at the Navajo Agricultural Products Industry (NAPI) and Navajo Mesa Farms during the 2017–2019 period. The collected adult pests were analyzed at Texas A & M University for the presence of *Candidatus* L. solanacearum (Lso). The results showed field infestation by *B. cockerelli* in early June and that the population peaked during the second half of July and decreased as the potato growing season progressed. However, a second less important peak of the pest was revealed around mid- to late-August, depending on the growing season and field. While the *B. cockerelli* population increased linearly with average air temperature, it showed strong third order polynomial relationships with the accumulated thermal units and the Julian days. The test of *B. cockerelli* for the Lso infection revealed a low incidence of the pathogen varying from 0.22% to 6.25% and the infected adult *B. cockerelli* were collected during the population peak period. The results of this study may be helpful to potato growers in pest management decision-making and control. However, more study is needed to evaluate zebra chip disease in terms of its prevention and economic impact, and to develop economic thresholds and pest management programs for northwestern New Mexico and neighboring regions.

## 1. Introduction

The potato psyllid, *Bactericera cockerelli* (Šulc; Hemiptera: Triozidae), is one of the most economically important pests for potato (*Solanum tuberosum* L.) production in North America [1,2]. Potato psyllids feed on the underside of leaves and suck within the potato vascular system, causing leaf chlorosis that impacts tuber yield of the potato crop [3]. Most importantly, *B. cockerelli* is recognized as a vector of the pathogenic bacterium, *Candidatus* Liberibacter solanacearum (Lso), also called Ca. Liberibacter psyllaurous, which causes the zebra chip disease [3,4,5,6,7,8].

Potato psyllid is indigenous to northern Mexico and the southwestern United States and migrates north mostly when climatic conditions are favorable [9,10]. Native and planted host plants of potato psyllid are mostly Solanaceae, Convolulaceae and Menthaceae families [11,12]. *B. cockerelli* has been found in Texas, Nebraska, Colorado, Kansas, California, New Mexico [13,14,15,16,17], Idaho, Washington, Oregon [6,18,19], Minnesota, Wisconsin, and North Dakota [20]. The population dynamics of *B. cockerelli* abundance vary spatially and temporally across the infested environment [16,21]. Using yellow sticky traps to capture the adult potato psyllid, Wenninger et al. [21] reported that *B. cockerelli* appeared in late May–early June and the population showed two peaks annually with the greatest peak in late August corresponding to the accumulated degree days of 1775 and 1498 °C in the Treasure and Magic valleys, respectively, in Idaho. Horton et al. [22] reported the first detection of the potato psyllid in small numbers during the last week of May and a significant peak in the late season in Idaho, Oregon and Washington, while it used to appear in the potato field in late June or early July in these states [23,24]. Swisher et al. [25] found the potato psyllid in Farmington (NM) to be native to northwestern New Mexico and that the decline in its population in late growing season might be due to unfavorable weather conditions, basically cold temperatures [17].

Crop production in northwestern New Mexico is entirely irrigated through river diversions distributed among the agri-business company Navajo Agricultural Products Industry (NAPI) and hundreds of small-scale producers. The NAPI farm is irrigated through the Navajo Indian Irrigation Project (NIIP) canal on a mesa top south of Farmington, NM and consists of more than 30,000 ha of cropland equipped with center pivot irrigation systems. The main crop species produced by NAPI are alfalfa, corn, wheat, beans, pumpkins and potatoes. Table stock potato cultivation represents a large part of the production system within the NAPI farm. Navajo Mesa Farms is a contractor producing exclusively chip potatoes on several hundred hectares of land leased from NAPI, having considerable impact on the chip potato industry. While the best agricultural practices are adopted to optimize resource efficiencies under potato production, insect pests could constitute a serious problem as most dangerous phytophagous ones are usually located at the lower side of potato leaves, reducing the efficacy of the contact insecticides [23,26,27]. More importantly, some pests are vector disease carriers with detrimental effects on potato yield and the physical and nutritional quality of the harvested potato tuber, including the zebra chip diseased transmitted by *B. cockerelli* to the potato plant. The monitoring of the *B. cockerelli* population and its dynamics through the potato growing season will help in timely control and management of the pest. The objectives of this study were to monitor the population dynamics of the potato psyllid and to test the captured psyllids for their associated pathogen *Candidatus* Liberibacter solanacearum (Lso), which represents the risk of zebra chip disease in northwestern New Mexico.

## 2. Materials and Methods

**Figure 1:**
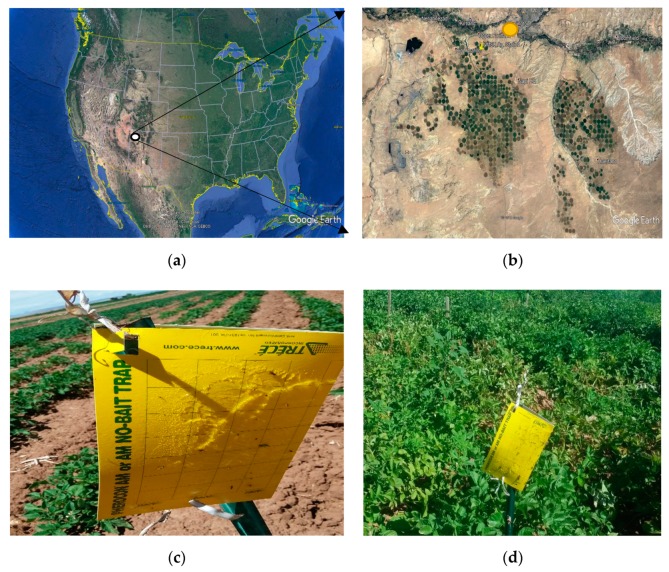
(**a**) Field locations of psyllid trapping at Farmington in Northwest New Mexico (white circle) in which (**b**) green circles represent the Navajo Agricultural Products Industry farms (Center pivot irrigated fields) where NAPI grows table potatoes and other different crops and Navajo Mesa grows chip potatoes; the yellow circle represents location of NMSU experiment station; (**c**,**d**) show sticky trap during the early season and the late season, respectively, with difference in vine development.

The current study was conducted at the New Mexico State University (NMSU) Agricultural Science Center at Farmington (Latitude 36.69′ North, Longitude 108.31′ West, Elevation 1720 m) (Figure 1a,b). The NMSU Agricultural Science Center is located within the NAPI farm and a part of this study was conducted in NAPI’s plots during the 2017 to 2019 growing seasons. NAPI grows table potatoes while the chip potatoes are grown by Navajo Mesa Farms, a contractor within NAPI. Weather variables were monitored at the experimental station by an automated weather station. Monthly average weather conditions for 2017, 2018, and 2019 are summarized in Table 1.

Insect traps used for the current study were standard unbaited yellow sticky traps (Trécé Pherocon AM) (Trécé Inc., Adair, OK, USA). The sticky traps were used for the direct monitoring of potato psyllids (*Bactericera cockerelli*) in the potato fields wherein 5 traps per field, covering a monitoring zone of about 10 m wide by 80 m long, evenly spaced in one block running N/S, were installed at a height of about 1 m (Figure 1c,d). While the field size at NMSU Agricultural Science Center is about 20 m by 90 m, the field size at NAPI is about a minimum of 50 hectares. Visual monitoring of insect damage to potato crops around the sticky traps was conducted weekly for control purposes (data not presented). This included psyllid-induced yellowing and/or presence of potato psyllid nymphs on the underside of potato leaves Adult *B. cockerelli* count was tallied weekly. Incidence of large numbers of *B. cockerelli* was used in decision-making on need for insecticide application on the commercial fields at NAPI and/or Navajo Mesa Farm. Data were collected during the active growing season, generally from early/late May through late August/early September.

For the analysis of the adult *Bactericera cockerelli* for *Candidatus* Liberibacter solanacearum (Lso) infection which causes potato zebra chip disease, the collected insects were sent for Lso analysis by Texas A & M AgriLife Center, Weslaco, Texas. Adult *B. cockerelli* collected were transferred into 70% ethanol before testing for Lso. The presence of Lso in *B. cockerelli* was determined by Polymerase Chain Reaction (PCR). The details of the analysis are presented in Munyaneza et al. [4], Wenninger et al. [21], Workneh et al. [17], and Harrison et al. [28]. The percentage of adult *B. cockerelli* testing positive for Lso was determined as the ratio of the number of *B. cockerelli* positive for Lso to the total number of collected *B. cockerelli* during each week of the monitoring period of each year.

Data were analyzed using CoStat statistical software. Three-way ANOVA was performed with the main effects as year (2017 to 2019), date (two-week intervals from June 1st to September 15th), the accumulated thermal units and their interactions. All *p* values ≤0.05 were considered statistically significant. The means were separated using Tukey’s test at the 95% level of probability to identify significant differences between seasonal total collected adult *B. cockerelli* per year. Regression analysis was also performed to develop the relationships between the adult of *B. cockerelli* counts and the average air temperature, the Julian day, and the accumulated thermal units. The coefficient of determination R^2^ was used to quantify the fitness of the relationships.

## 3. Results and Discussion

### 3.1. Variation in the Abundance of the Adult of B. Cockerelli during the Potato Growing Season

**Figure 2:**
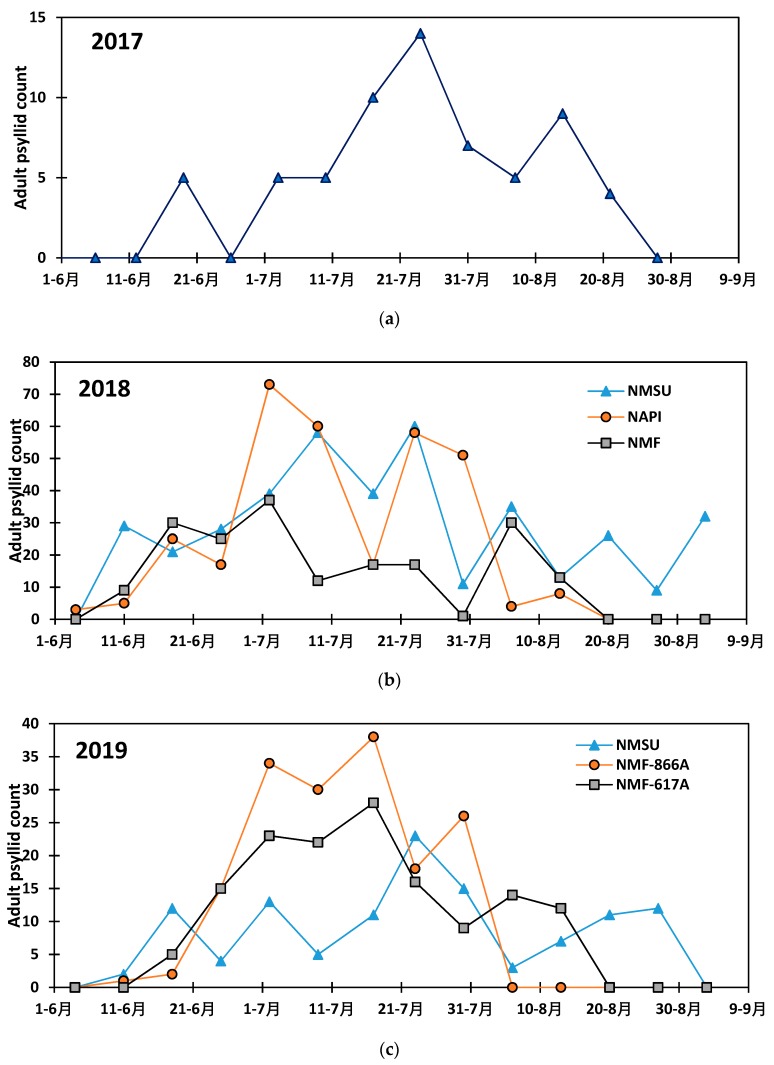
Population dynamics of *Bactericera cockerelli* in the study area during the (**a**) 2017, (**b**) 2018, and (**c**) 2019 potato growing seasons.

Seasonal occurrence of adult potato psyllid is presented in Figure 2, and during the 2017, 2018 and 2019 growing seasons, respectively. The statistical analysis showed that the abundance of *B. cockerelli* varied with years (*p* < 0.0001) and the trapping date (*p* = 0.0138). The pest was more abundant during the 2018 growing season than in 2019 and 2017 growing seasons with a weekly average adult *B. cockerelli* collection of 65, while only 30 and 5 adult *B. cockerelli* were collected weekly on average during the 2019 and 2017 growing seasons, respectively. The comparison of the number of adult *B. cockerelli* collected weekly throughout the potato growing season revealed the highest insect population during July with non-significant differences between the numbers of *B. cockerelli* collected weekly during the month. On the biweekly basis, the population of *B. cockerelli* varied with years (*p* = 0.0076) and the adult trapping period (*p* = 0.0001).

Across the study area, adult *B. cockerelli* appears at the beginning of June and the population increases and peaks in mid to late July and decreases toward the end of August−early September. During the 2017 growing season, *B. cockerelli* was collected only at the NMSU research station and the population increased from June 11 to July 24 with a peak population of 14 adults of *B. cockerelli* collected and decreased thereafter. After potato vine killing by the end of August, the *B. cockerelli* disappeared from the plot due the lack of the host potato plant. During the 2018 growing season, adults of *B. cockerelli* appeared at the beginning of June and the population increased and peaked in July at the NMSU field with 58 adults *B. cockerelli* collected a week while the peak population was found in late June early July with 73 adults *B. cockerelli* collected, and peak was shown in early July with 12 adults collected a week (Figure 2). However, the abundance of *B. cockerelli* showed a second population peak occurring in late July at NAPI farm and early August in the Navajo Mesa farm and at NMSU. During the 2019 growing season, similar population trend was observed across all three fields. As previously observed in 2017 and 2018, *B. cockerelli* appeared during the second week of June and its number increased up to a peak of 38 adults in one of the Navajo Mesa field, 28 adults in the second field of Navajo Mesa, and 23 adults at NMSU during the third week of July (Figure 2). The abundance of *B. cockerelli* decreased thereafter toward the end of August−early September which coincided with potato vine killing period.

**Figure 3:**
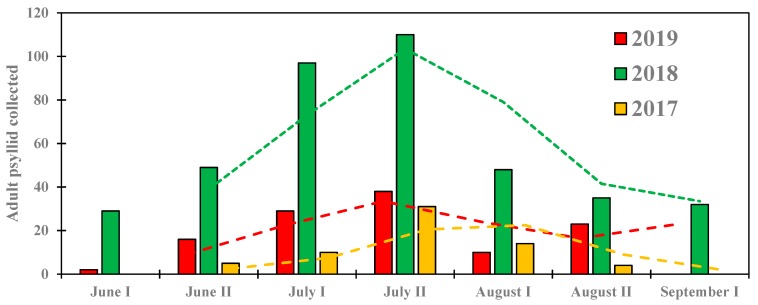
Biweekly abundance of the total adults of *B. cockerelli* collected across all experimental fields during the 2017, 2018, and 2019 potato growing seasons.

Combining adults’ data of all fields on a two-week basis during the 2017 growing season, the first catch of adult *B. cockerelli* occurred during the second half of June and the population peaked during the second half of July with a total of 31 adults cached (Figure 3). During the 2018 growing season, adults appeared earlier than during the 2017 growing season. The population increased early June from 29 collected adults to 110 adults during the second half of July and decreased to 32 adults collected during the first half of September. During the 2019 growing season the population dropped as compared to the 2018 growing season *B. cockerelli* appeared early June and its population increased from two adults early June to 38 during the second half of July and decreased thereafter. As presented in Figure 3, the moving average of two-week period showed the *B. cockerelli* population peak occurring consistently during the second half of July in 2017, 2018, and 2019.

The results of this study are in agreement with some previous studies on the populations dynamics of the potato psyllids. Munyaneza et al. [23] reported that *B. cockerelli* appeared late July and the population peak magnitude and timing varied with years and occurred during the period of late August to the third week of September in Yakima and Benton counties in Washington State. Seasonal variation in potato psyllid population was also reported by Goolsby et al. [16] who indicated the variation of *B. cockerelli* population across years in potato growing regions in Texas, Nebraska and Colorado. Similar trend was reported in Texas and New Mexico by Workneh et al. [17]. Teresani et al. [29] collected the maximum psyllid species during summer months with the different peak magnitudes occurring in July at some locations and in August at other locations in Spain and the Canary Islands. *B. cockerelli* appeared in Washington State and Oregon in late June and early July [6,30]. Similarly, Randon et al. [24] reported the potato field invasion by *B. cockerelli* during late June to Late July across the Pacific Northwest United States. The results of the present study are opposed to the findings of Randon et al. [24] who reported that *B. cockerelli* was not detected before mid-July and its population steadily increased throughout the potato growing season in Columbia Basin (Oregon). Horton et al. [31] also reported that *B. cockerelli* was in a low population from late May to late June and its number continued to increase from mid−July as the season progresses up to potato harvest in September in Washington State. Similar findings were reported by Wenninger et al. [32] in the southern Idaho. The results of the temporal variation in *B. cockerelli* population in the Four Corners region should aid in planning for the control of the pest as a very large acreage of table and chip potato is grown every season in the region and which is of a prime economic importance for the commercial potato growers and the small scale producers.

### 3.2. Impact of Air Temperature and Thermal Unit on the Abundance of Bactericera Cockerelli during the Potato Growing Season

**Figure 4:**
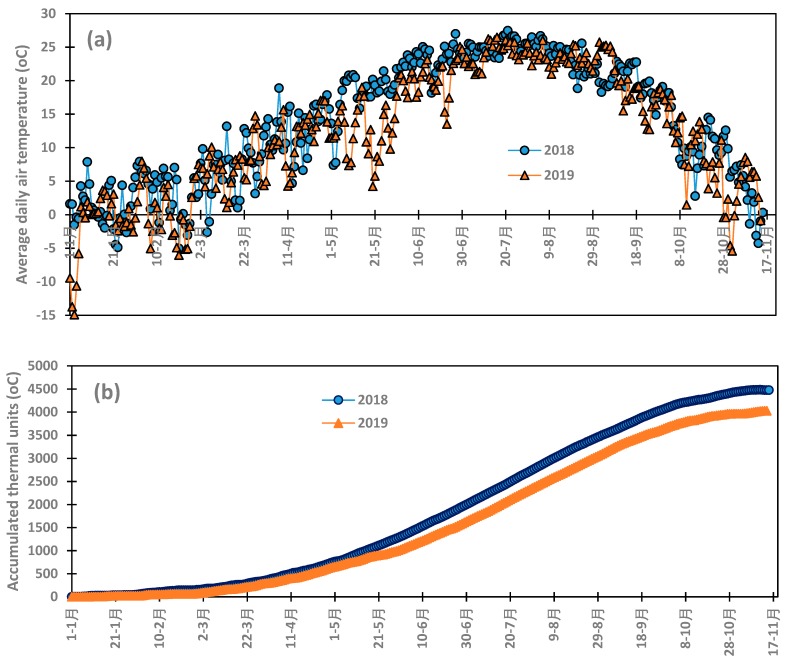
Evolution of air temperature (**a**) and the cumulated heat units (**b**) from January 1st to November 15th during 2018 and 2019.

Average air temperature during the year 2018 and 2019 is presented in Figure 4. The Figure 4a shows the cumulative heat units from January 1st to November 15th of the years 2018 and 2019. Due to technical problem, climatic parameters were not collected during a part of the year 2017 with is not considered for the analysis in this section. Air temperature increased from January to the maximum of 26 °C 28 °C at the end of July 2018 and 2019, respectively. The 2018 season was warmer than 2019 and the difference in the accumulated heat unit is shown in Figure 4b. The total heat units accumulated in 2018 was 4477 °C while the total was 4026 °C in 2019, representing a 10% decrease in the total heat units.

**Figure 5:**
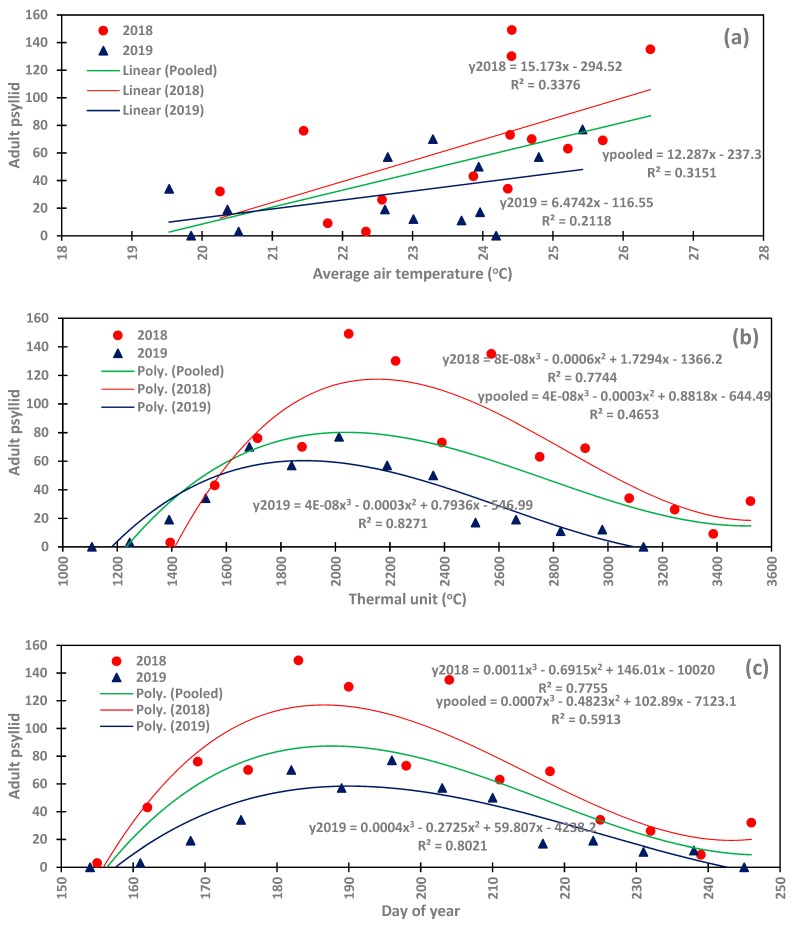
Correlation between the abundance of *Bactericera cockerelli* and (**a**) average air temperature; (**b**) accumulated thermal unit; and (**c**) Julian day during 2018 and 2019.

Abundance of *B. cockerelli* was linearly correlated to average air temperature (Figure 5a). The population of the pest increased as average air temperature increased, and the slope of the regression varied with years and was 15.17 in 2018 band 6.47 in 2019 while the coefficient of determination R^2^ of the correlations was 0.34 in 2018 and 0.21 in 2019. R^2^ values are relatively low and expressed on average the weakness of the impact of air temperature on the dynamics of the population of *B. cockerelli* in the Four Corners region. The 2018 and 2019 pooled data showed also positive linear relationship with air temperature with the regression slope of 12.29 and coefficient of determination R^2^ of 0.32. Potato psyllid appeared when average air temperature is greater than 19 °C during both potato growing seasons. The abundance of potato psyllid showed better relationship with the accumulated thermal unit compared to its relationship with average air temperature.

Potato psyllid population had strong third order polynomial correlation with the accumulated thermal unit in 2018 and 2019 with the R^2^ values of 0.77 and 0.83 in 2018 and 2019, respectively and the pooled data also showed similar correlation with R^2^ value of 0.46 (Figure 5b). The lower R^2^ value of the pooled data correlation is due seasonal or annual variation in the abundance of the *B. cockerelli*. The presence of potato psyllid in the study area started with the accumulated thermal unit about 1400 °C in both 2018 and 2019. Potato psyllid population increased as the thermal unit increased and peaked at the thermal unit values of 2049 °C and 2013 °C in 2018 and 2019, respectively. The population decreased thereafter to the minimum around 3387 °C in 2018 and 3130 °C in 2019.

The population of *B. cockerelli* during potato growing season showed strong third order polynomial relationship with the Julian day as shown in Figure 5c. The coefficients of determination of the relationships between the dynamics of the potato psyllid population and the Julian day were 0.78 and 0.80 for the 2018 and 2019, growing seasons, respectively, while the 2018 and 2019 pooled data had a lower coefficient of determination R^2^ value of 0.59 due to the difference in the magnitude of the abundance of the pest during the two seasons.

The relationships developed in the study are indicative of the occurrence and the magnitude of the abundance of potato psyllid in the northwestern New Mexico. While there is a difference in the peaks, the start point was the same around the value of 1390 °C of the accumulated thermal unit. This start point may not coincide with the same Julian day from one year to the other because average air temperature usually varies with years as 2018 was warmer than 2019 (Figure 4b).

The results of the present study corroborate with the finding of Antolínez et al. [33] who reported the arrival of potato psyllid in the potato fields when the average air temperature is greater than 15 °C and the peak population occurred when air temperature was about 25 °C in Spain. Wenninger et al. [21] also correlated the population abundance of adults *B. cockerelli* with the accumulated thermal unit and found the population peak at 1775 °C in late August in the Treasure Valley and at 1498 °C in the Magic Valley in Idaho. Lewis et al. [34] suggested integrating accumulated thermal units into the management and control of *B. cockerelli* and proposed a model that integrates thermal unit to simulate the development of potato psyllid infestation.

### 3.3. Bactericera Cockerelli Infection by Zebra Chip Pathogen (Candidatus Liberibacter Solanacearum)

**Figure 6:**
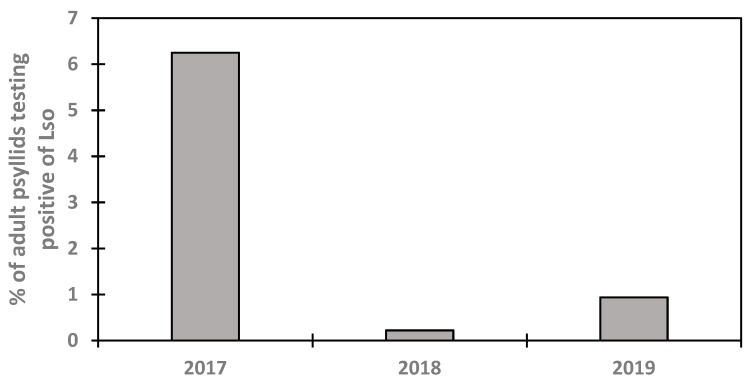
Percentage of infected adult psyllids collected in sticky traps during the 2017−2019 period.

The infection rating of *Bactericera cockerelli* by zebra chip disease pathogen *Candidatus* Liberibacter solanacearum (Lso) was revealed as low during the study period (Figure 6). The infected adults of the potato psyllid were collected during the population peak period around mid-July to late July. During the 2017 monitoring period, there were four adults *B. cockerelli* infected with the pathogen over a total of 64 adults of potato psyllid collected and which represent 6.25% of the total adults collected during the 2017 season. During the 2018 monitoring period, a total of 912 adult *B. cockerelli* were collected and only two adults were test positive to Lso, representing 0.22% of infected adults. During the 2019 monitoring season, there were four adults *B. cockerelli* tested positive to Lso over a total of 426. This rating represents 0.94% of the total adults *B. cockerelli* collected during the 2019 season. These results are in agreement with those of similar studies conducted across the United States. Workneh et al. [17] reported as low as 11 adults of *B. cockerelli* tested positive over about 1800 *B.* cockerelli collected during four-year study period across Texas and at Farmington, NM. Goolsby et al. [16] reported infection rating of *B. cockerelli* by Lso below 8% of the total trapped *B. cockerelli*, and which varied with years and locations across Texas, Kansas, Colorado, and Nebraska. The percentage of the total adult *B. cockerelli* tested positive if Lso was below 1% in Kansas and Nebraska while it greatly varied across Texas with higher infection rating [16]. Wenninger et al. [35] also reported a low incidence of *Candidatus Liberibacter solanacearum* over four years while a high infection rate of 23% was shown in one year during the 2013−2017 study. The results of this study should be considered for decision making regarding timely pest management as Goolsby et al. [16] reported a strong positive correlation between the infected adult psyllids and zebra chip disease incidence in harvested potato tubers. A previous study by Workneh et al. [17] demonstrated that the Southern *B. cockerelli* haplotype (lower Rio Grande Valley, Texas) are the dominant one monitored in Farmington, NM during the 2014−2017 period. Further, with the high infection rating of the *B. cockerelli* in Texas [16], there should be a rigorous surveillance and monitoring program for the potato psyllid control to reduce the incidence of zebra chip disease in the commercial potato field at NAPI and Navajo Mesa farms and in the small-scale farms. While Walker et al. [36] reported an economic threshold of three adults of potato psyllid per sticky trap, Randon et al. [24] indicated that there is no population economic threshold for the potato psyllid and action should be taken once the pest is detected in the field and Munyaneza [2] indicated that the potato psyllid controls start at plant emergence in the Southwest due to the presence of the pest at crop emergence.

## 4. Conclusions

The population dynamics of the potato psyllid (*Bactericera cockerelli*) were monitored and the infections of adults were tested for zebra chip disease pathogen *Candidatus* Liberibacter solanacearum that puts the potato production at risk of the disease in northwestern New Mexico. Overall, adults of potato psyllid started infesting the potato farms in early June and the pest population peaked during the second half of July, with a second les important peak thereafter. *B. cockerelli* population showed strong third order polynomial relationships with the accumulated thermal unit and Julian days with a high coefficient of determination R^2^ value for each year ≥0.77. Meanwhile, the pooled data showed lower R^2^ due to seasonal variation in the abundance of the pest. A *Candidatus* Liberibacter solanacearum infection test revealed a low incidence of the pathogen varying from 0.22% to 6.25% of adult *B. cockerelli* and the pathogen is present during the population peak of *B. cockerelli*. While the results of this study might help commercial potato growers and small scale growers in pest management, further study must be conducted to evaluate the zebra chip disease and develop an economic threshold and pest management program to reduce the economic impact of the disease on potato production.

## Figures and Tables

**Table 1:** Weather conditions during the 2017–2019 period at NMSU Experiment Station at Farmington.

**2017**									
**Months**	**Tmax (°C)**	**Tmin (°C)**	**Tmean (°C)**	**RHmax (%)**	**RHmin (%)**	**RHmean (%)**	**U_2_ (m/s)**	**Rs (MJ/m^2^)**	**Precip (mm)**
January	5.6	−4.8	0.3	0.5	0.2	0.3	2.0	8.1	10.4
February	12.3	−0.7	4.6	0.5	0.2	0.3	1.7	11.1	6.1
March	16.8	1.5	9.0	80.1	21.4	50.8	3.6	16.2	8.9
April	18.4	1.1	10.1	72.8	15.8	41.0	2.6	22.3	37.1
May	23.1	5.8	14.5	71.5	15.5	40.4	2.6	25.4	22.6
June	32.5	12.4	23.2	56.6	6.8	24.0	2.2	30.2	0.0
July	32.9	16.5	24.3	75.8	18.4	44.8	2.0	26.7	47.5
August	30.6	14.5	22.5	73.4	17.5	41.8	1.8	23.3	1.8
September	27.2	10.6	18.5	72.0	19.7	43.0	2.0	20.1	46.0
October	21.1	2.2	11.2	63.5	13.6	34.6	2.0	16.4	0.0
November	17.1	0.1	8.0	68.0	19.8	41.6	1.8	11.4	2.8
December	10.4	−6.9	0.9	61.2	17.3	38.0	1.8	10.6	0.0
**2018**									
**Months**	**Tmax**	**Tmin**	**Tmean**	**RHmax**	**RHmin**	**RHmean**	**U_2_**	**Rs**	**Precip**
January	9.7	−6.1	0.9	76.5	24.6	50.2	2.0	11.6	5.6
February	11.5	−4.3	3.2	74.9	19.4	44.8	2.6	13.5	1.3
March	15.3	−2.5	6.6	62.4	14.0	34.9	2.8	18.3	3.6
April	21.3	2.7	12.9	50.5	8.2	23.0	3.4	23.3	5.9
May	26.9	8.0	17.9	51.8	8.4	24.4	2.6	27.2	6.4
June	32.0	12.9	23.1	52.8	7.4	23.1	2.2	29.2	17.8
July	33.5	16.9	25.0	71.5	15.3	39.3	2.2	26.9	11.7
August	31.7	15.6	23.6	66.1	13.5	35.5	1.9	23.4	3.6
September	28.5	11.6	19.8	65.8	15.2	35.4	1.6	21.1	3.0
October	17.1	3.2	9.8	89.3	32.7	60.7	2.1	13.3	10.7
November	10.8	−5.6	2.2	70.5	21.6	43.6	1.8	12.6	1.3
December	5.5	−6.1	−0.7	87.3	42.6	67.6	1.9	8.8	9.1
**2019**									
**Months**	**Tmax**	**Tmin**	**Tmean**	**RHmax**	**RHmin**	**RHmean**	**U_2_**	**Rs**	**Precip**
January	5.0	−7.0	−1.5	94.3	45.3	72.9	2.0	10.2	12.7
February	7.2	−5.6	0.4	87.3	33.5	62.5	2.4	12.3	21.6
March	14.2	0.1	7.0	83.5	26.2	53.9	2.7	17.2	34.5
April	19.5	3.0	11.7	71.8	17.1	39.9	2.8	21.6	6.6
May	19.8	4.3	12.3	82.5	23.2	50.3	2.3	23.1	43.4
June	28.9	11.1	20.4	70.6	12.9	35.4	2.3	27.2	5.8
July	32.3	15.2	24.0	70.3	14.5	37.0	1.9	26.8	7.1
August	32.0	14.6	23.2	68.2	15.3	36.9	1.6	24.3	1.5
September	27.6	11.3	19.3	73.8	18.6	43.3	2.2	20.3	9.9
October	15.7	−2.8	6.5	52.7	14.3	30.9	2.1	16.9	2.3
November	15.5	−3.6	5.5	66.5	14.5	35.7	1.5	14.3	0.0

U_2_ is wind speed measured at 2 m; Rs is solar radiation.
